# SVR Rates of HCV-infected population under PEG-IFN-α/R treatment in Northwest China

**DOI:** 10.1186/s12985-017-0708-6

**Published:** 2017-03-23

**Authors:** Yanhua Li, Jiuping Wang, Juan Wang, Yunfeng Xiao, Bin Xu, Hongwei Li, Liu Yang, Xiaoke Hao, Yueyun Ma

**Affiliations:** 1 Department of Clinical Laboratory Medicine, Xijing Hospital, The Fourth Military Medical University, 169 Changle West Road, Xi’an, Shaanxi 710032 People’s Republic of China; 2 Department of Infectious Disease, Xijing Hospital, Fourth Military Medical University, Xi’an, Shaanxi 710032 People’s Republic of China; 3 Pharmacy Department, Tangdu Hospital, The Fourth Military Medical University, Xi’an, Shaanxi 710038 People’s Republic of China; 4 Shandong International Trust Co., Ltd, Jinan, Shandong 250013 People’s Republic of China

**Keywords:** Chronic hepatitis C, Interleukin 28, Single nucleotide polymorphism, HCV subtype, Cirrhosis, Individualization

## Abstract

**Background:**

Chronic HCV Patients taking PEG-IFN-α/R from different ethnic groups have different probabilities of reaching a sustained viral response (SVR). There are many influence factors, such as HCV genotype, IL-28B single-nucleotide polymorphisms (SNP), Fibrosis 4 index (FIB-4), and aspartate aminotransferase-to-platelet ratio index (APRI) score. But the baseline factors in relation to treatment outcome was still not much clear.

**Methods:**

We evaluated data from 231 chronic HCV patients with or without liver fibrosis and their antiviral efficacy after treatment with pegylated interferon plus ribavirin (PEG-IFN-α/R) for 24–48 weeks. IL-28B SNP and HCV genotypes were analyzed with genome sequencing using pyrosequencing.

**Results:**

Sustained viral response (SVR) rates of patients with HCV 1b and 2a genotypes were 52.25% (58/111) and 75.28% (67/89) (P < 0.01). SVR rates of patients with IL-28B rs8099917 TT, rs12979860 CC and rs12980275 AA were 92.41% (25/27), 92.86% (26/28) and 88.89% (24/27) separately. We found that SVR rates in HCV 1b and 2a patients were only 31.0 and 39.4% if their FIB-4 > 3.25. In addition, when their APRI > 2, only 30.3% of HCV 1b patients and 50.2% of HCV 2a patients could obtain SVR.

**Conclusions:**

There were high proportion of HCV genotype 1b and 2a in Northwest China. In both HCV 1b and 2a genotypes, patients with protective-genotype of IL-28B were more likely to obtain SVR. However, those with significant fibrosis or cirrhosis were less likely, no matter their genotype. Combined factors of HCV genotype, IL-28B genotype, FIB-4 and ARPI may indicate high prediction and clinical value regarding treatment with PEG-IFN-α/R and prognostic evaluation of chronic hepatitis C patients.

## Background

Hepatitis C is an infectious disease that is widely spread geographically. It has been reported that about 180 million people are infected with the hepatitis C virus (HCV) worldwide, accounting for about 3% of the current population [1]. The incidence of infection can be easily neglected and thus may develop into cirrhosis and even HCV-related hepatocarcinoma and liver failure, which pose serious threats to human health. More than 0.35 million people have died from HCV-related liver disease [2]. The application of pegylated interferon (PEG-IFN)-alpha combined with ribavirin (PEG-IFN-α/R) had been considered to be the most popular and effective therapy in blocking virus replication before 2015 [3]. While the novel sofosbuvir-ledipasvir opened a new time for treatment of eligible HCV-infected patients with 90–100% efficiency [4, 5]. Unfortunately sofosbuvir is much more expensive, with an estimated cost of an additional $65 billion during the next 5 years [6]. It should be carefully thought about the necessity and how much people have to take DAA drugs. And it is not the time to ignore the application of PEG-IFN-α/R, especially in China.

Patients taking PEG-IFN-α/R from different ethnic groups have different probabilities of reaching a sustained viral response (SVR). In the United States, the rate of obtaining SVR in the black population is almost 50% less than of white patients, and the probability of obtaining SVR in white patients with HCV genotype 1 treated by PEG-IFN-α/R is approximately 42–53% [7]. In China, the SVR rate was a light higher, could reach 65.3% [8]. We recently found that northwest patients of China seem to have higher SVR that was 72.6%. Because there were seldom immigrant in the northwest population, the high SVR is reasonable caused by geographic specificity, such as genotype of HCV and polymorphisms of IL-28B.

Determination of HCV genotype is important to predict response to antiviral therapy and time of treatment [9]. Early clinical trials have reported that SVR rates for patients with genotype 1 (42–46%) are lower than rates for patients with non-type 1 genotype (76–82%) [10]. On the other hand, genome-wide association studies have suggested that host IL-28B single-nucleotide polymorphisms (SNP) rs12979860 CC and rs8099917 TT are significantly correlated with SVR in patients receiving PEG-IFN-α/R treatment [11]. The probability of obtaining SVR is further reduced in vivo if IL-28B is mutated in patients with HCV genotype 1 infection [12, 13].

Liver fibrosis had been suggested being closely associated with the risk of HCC development in chronic hepatitis C patients [14]. The eradication of HCV with antiviral therapy will prevent the progression of chronic hepatitis and associated complications [15]. But it was never paid more attention in China [8].

To determine the favorable patients for treatment with PEG-IFN-α/R, HCV genotype, IL-28B SNP, Fibrosis 4 (FIB-4) index and aminotransferase-to-platelet ratio index (APRI) score we retrospectively analyzed with SVR in this study.

## Methods

### Patients

All patients with CHC were enrolled for this research with signed informed consents following the protocols approved by the Institutional Review Board of the Fourth Military Medical University (Table 1). Inclusion criteria: patients were firstly diagnosed as HCV infection since August 2013, naive-treatment from October 2013 and HCV RNA > 1000 IU/mL. Exclusion criteria: Patients with recurrence of hepatitis C, hepatitis infected with HAV, HBV, HDV, HEV, EBV or CMV; HIV infection, Diabetes, autoimmune liver disease and HCC etc. were excluded.

**Table 1:** Clinical characteristic of patients with different HCV genotypes

HCV Genotype	No.	Age (y), range	Sex (No. of Patients, %)		Viral load (10^6^ IU/mL), range	ALT (U/L), range	AST (U/L), range
			Male	Female			
1a	12	39.67 (24–60)	7 (58.33)	5 (41.67)	2.33 (0.01–29.90)	58.17 (19–249)	37.08 (10–157)
1b	111	46.81 (20–79)	52 (47.27)	58 (52.73)	1.62 (0.02–46.1)	50.15 (13–392)	44.96 (16–453)
2a	89	50.45 (22–80)	40 (44.94)	49 (55.06)	2.60 (0.00–18.10)	49.38 (12–554)	35.25 (15–205)
2b	1	54.00 (54–54)	0 (100.00)	1 (0.00)	1.08	286.00	351.00
3a	11	37.91 (25–53)	8 (72.73)	3 (27.27)	5.52 (0.21–29.5)	91.27 (37–340)	50.36 (32–160)
3b	5	38.00 (23–45)	3 (60.00)	2 (40.00)	1.71 (0.10–6.88)	52.40 (58–204)	37.00 (44–141)
4a	0						
5a	1	43.00 (43–43)	2 (100.00)	0 (0.00)	0.00	0.00	0.00
6a	1	25.00 (25–25)	1 (100.00)	0 (0.00)	0.00	0.00	0.00
		*Z* = 3.6464	chi-squared =0.643		*Z* = 1.8797	*Z* = 0.6820	*Z* = 0.6820
*P* value		.067	.836		.113	.605	.605

The low number of patients with 2b, 4a, 5a and 6a did not meet the statistical requirements; therefore, these were excluded for follow-up statistics

We collected data on 230 patients with chronic HCV infection who were seen at Xijing Hospital from October 2013 to February 2016. The average age was 46.71 years (range, 20–80 y), with 112 male and 118 female patients. Patients had been treated with standard of care for 24–48 weeks. PEG IFN-α/RBV: The recommended dose of PEG IFN-α-2a (Pegasys Roche Shanghai) for chronic hepatitis C was 180 ug per time, once a week, subcutaneous injection of the abdomen or thigh. The dose of RBV was determined by the genotype of virus: the dose for genotype 2 or 3 was 800 mg a day for 24 weeks, and the dose of genotype 1 was 1000–1200 mg daily according to body weight, for 48 weeks. We mainly investigated outcomes after 24 weeks.

There are many serological markers for HCV or evidence of liver disease including HCV RNA was used to understand the activity of virus replication, ALT, AST, Total bilirubin, direct bilirubin, indirect bilirubin, albumin, globulin, choline esterase, alkaline phosphatase, phosphatase and abdominal ultrasound were used for evaluation of liver damage, CT or MRI was used to be clear of the extent of liver damage. Liver biopsy is the gold standard for evaluation of liver inflammation and fibrosis staging in patients. In addition, there were also patient compliance issues. After careful consideration we chose these common and easy to get markers as ALT and AST for liver damage, FIB-4 and APRI to evaluate liver fibrosis. Use of viral content to evaluate curative effect was according to the 2014 European Association for the Study of the Liver Recommendations on Treatment of Hepatitis C and the Guideline of Prevention and Treatment of Hepatitis C [16, 17]. We evaluated SVR using quantitative real-time fluorescence polymerase chain reaction (ViiA7 OX, Life Technology) of HCV RNA (less than 15 IU/mL) for at least 24 weeks of follow-up at the end of treatment. Samples were collected and separated from the peripheral blood and serum and then stored at −20 °C until analyses.

### DNA/RNA extraction

For DNA extraction for IL-28B gene detection, we used a blood genomic DNA extraction kit (Tiangen, Beijing, China). For HCV RNA extraction, we used the MinElute column QIAamp method and the virus genome DNA/RNA extraction kit (Tiangen). All extracted DNA/RNA were then immediately used for gene detection or stored at −80 °C.

### Quantitative real-time fluorescence PCR

Primer sequences were designed by using DNAMan 6.0.3.99 (LynnonBiosoft, San Ramon, CA, US) for IL-28B gene amplification, including IL-28B rs8099917, IL-28B rs12979860 and IL-28B rs12980275 genes (Table 2). Among them, 5’of 3reversed primers were labeled with biotin. Amplification included heating at 95 °C for 5 min, 95°Cfor 20 s, and 60 °C for 20 s, with steps repeated for 40 cycles. Amplification was run on a 7500 fast real-time PCR system (Applied Biosystems, Foster City, CA, USA). HCV genotype was measured by using an HCV genotyping PCR kit (Qiagen, Beijing, China), following the manufacturer’s protocol.

**Table 2:** Primers for genotype analysis of IL-28B

Primer	IL-28B	Sequence
RT-PCR primers	rs12989760 forward:	5′-GTCGTGCCTGTCGTGTACTGA-3′
	rs12989760 reverse:	5′-AGCGCGGAGTGCAATTCA-3′
	rs8099917 forward:	5′-CTCCTTTTGTTTTCCTTTCTGTGA-3′
	rs8099917 reverse:	5′-ACATAAAAAGCCAGCTACCAAACT-3′
	rs12980275 forward:	5′-ACATGAGGTGCTGAGAGAAGTCAA-3′
	rs12980275 reverse:	5′-TACCCCGGCAAATATTTAGACAC-3′
Sequencing primers	rs12989760	5′-AGCTCCCCGAAGGCG-3′
	rs8099917:	5′-TCCTTTCTGTGAGCAAT-3′
	rs12980275:	5′- GAAGTCAAATTCCTAGAAA −3′

### Gene sequencing

IL-28B gene polymorphism was detected with the use of a pyrosequencing method on Q24MDX (Qiagen, Hilden, Germany). Sequencing primers were designed by the Q24 PyroMark. HCV polymorphism sequencing primers were also provided by Qiagen.

### Liver fibrosis staging

The degree of liver fibrosis was evaluated with APRI score, which can be used for the assessment of liver cirrhosis [18]. APRI scores > 2 in adults indicate that the patient has already had liver cirrhosis. The APRI score is calculated as follows: (aspartate aminotransferase [AST]/ULN) × 100/platelets (10^9^/L), where ULN is the upper limit of normal value. Fibrosis-4 index was based on values of ALT, AST, platelet count and patient age. This index can be used to diagnose liver fibrosis (similar to least significant fibrosis using METAVIR F scoring system ≥2) [19]. A significant liver fibrosis has occurred if a patient shows aFIB-4 index of >3.25. FIB-4 is calculated as follows: age × ALT (IU/L)/(platelet count [10^9^/L] × AST [IU/L]) ^1/2^.

### Statistical analyses

We used Pearson chi-squared and Kruskal-Wallis tests to analyze the qualitative data with SPSS 19.0 (IBM, USA.). A logistic regression model was used to analyze the correlation between SNPs (IL-28B rs8099917, IL-28B rs12979860 and IL-28B rs12979860) and SVR of patients. Odds ratio (OR) was used to describe the correlating degree of disease and exposed factors. OR tests were two-sided tests, in which *P* < 0.05 was considered to be statistically significant.

## Results

### HCV genotype and distribution

**Fig. 1:**
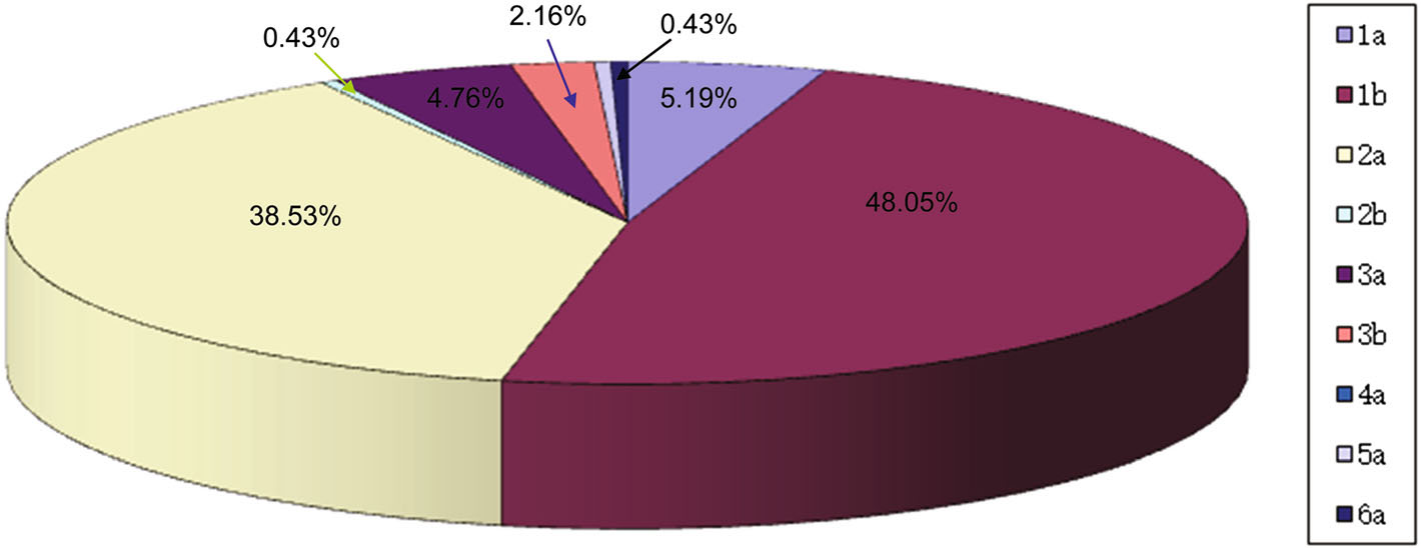
Distribution of HCV genotype. 86% of the patients with HCV infection were genotype 1 and 2, with 1b accounting for 48.05% and 2a accounting for 38.53%, followed by 1a (5.19%), 3a (4.76%), 3b (2.16%), 2b (0.43%), and 6a (0.43%)

HCV genotypes of 230 patients with chronic HCV infection were sequenced, with results shown in Table 2. One patient had mixed infection of 1b and 5a. For statistical analyses, this patient was analyzed in both the 1b and 5a genotype groups. Statistical results of 1a, 1b, 2a, 2b, 3a, 3b, 4a, 5a and 6a genotypes are presented in Fig. 1. The ratio of genotypes were as follows: 5.19% with 1a (12/230),48.05% with 1b (111/230),38.53% with 2a (89/230),0.43% with 2b (1/230),4.76% with 3a (11/230),2.16% with 3b (5/230),0% with 4a (0/230),0.43% with 5a (1/230), and 0.43% with 6a (1/230). There were no significant differences in age, sex, viral load and alanine aminotransferase (ALT) and aspartate transaminase (AST) levels (*P* > 0.05).

### IL-28B genotype in patients with chronic HCV infection

Fifty-one patients with HCV were included in the IL-28B gene polymorphism loci analyses (Table 3). Of the IL-28B genotypes, 76.47% of patients were rs8099917 TT, 76.47% were rs12979860 CC, and 72.55% were rs12980275 AA. We also found that 5.88% of the IL-28B genotypes were rs8099917 GG, rs12979860 TT or rs12980275 GG. Details of clinical features are presented in Table 3. Statistical results showed no statistical differences in age, sex, viral load and ALT and AST levels (*P* > 0.05).

**Table 3:** IL-28B gene polymorphism in patients with chronic HCV infection and clinical characteristics of patients with different IL-28B SNPs

Genotype	SNP	Percent	Age (y)	Sex (No. of Patients, %)		Viral Load (10^6^ IU/mL), range	ALT (U/L), range	AST (U/L), range
		(%)		Male	Female			
IL-28B rs8099917	TT (*n* = 39)	76.47	48 (25–64)	54 (52.9)	52 (47.1)	7.98 (0.24–72.46)	34 (18–179)	38 (17–203)
	TG + GG (*n* = 12)	23.43	47 (22–58)	21 (55.3)	16 (44.7)	9.25 (0.89–59.71)	36 (28–150)	40 (24–138)
IL-28B rs12979860	CC (*n* = 39)	76.47	49 (22–64)	57 (52.3)	52 (47.7)	10.23 (1.02–68.52)	35 (21–112)	32 (19–143)
	CT + TT (*n* = 12)	23.43	49 (20–58)	18 (52.9)	16 (47.1)	8.72 (0.48–65.32)	37 (19–135)	34 (17–178)
IL-28B rs12980275	AA (*n* = 37)	72.55	47 (28–64)	51 (51.5)	48 (48.5)	11.25 (1.37–66.68)	38 (22–144)	31 (20–123)
	AG + GG (*n* = 14)	27.45	46 (25–59)	24 (54.5)	20 (45.5)	9.67 (0.69–71.26)	32 (19–179)	29 (17–112)
			*Z* = 0.125	chi-squared = 0.413		*Z* = 0.579	*Z* = 0.378	*Z* = 2.143
*P* value			.912	.648		.825	.617	.346

### Correlation between HCV genotypes and antiviral efficacy

**Fig. 2:**
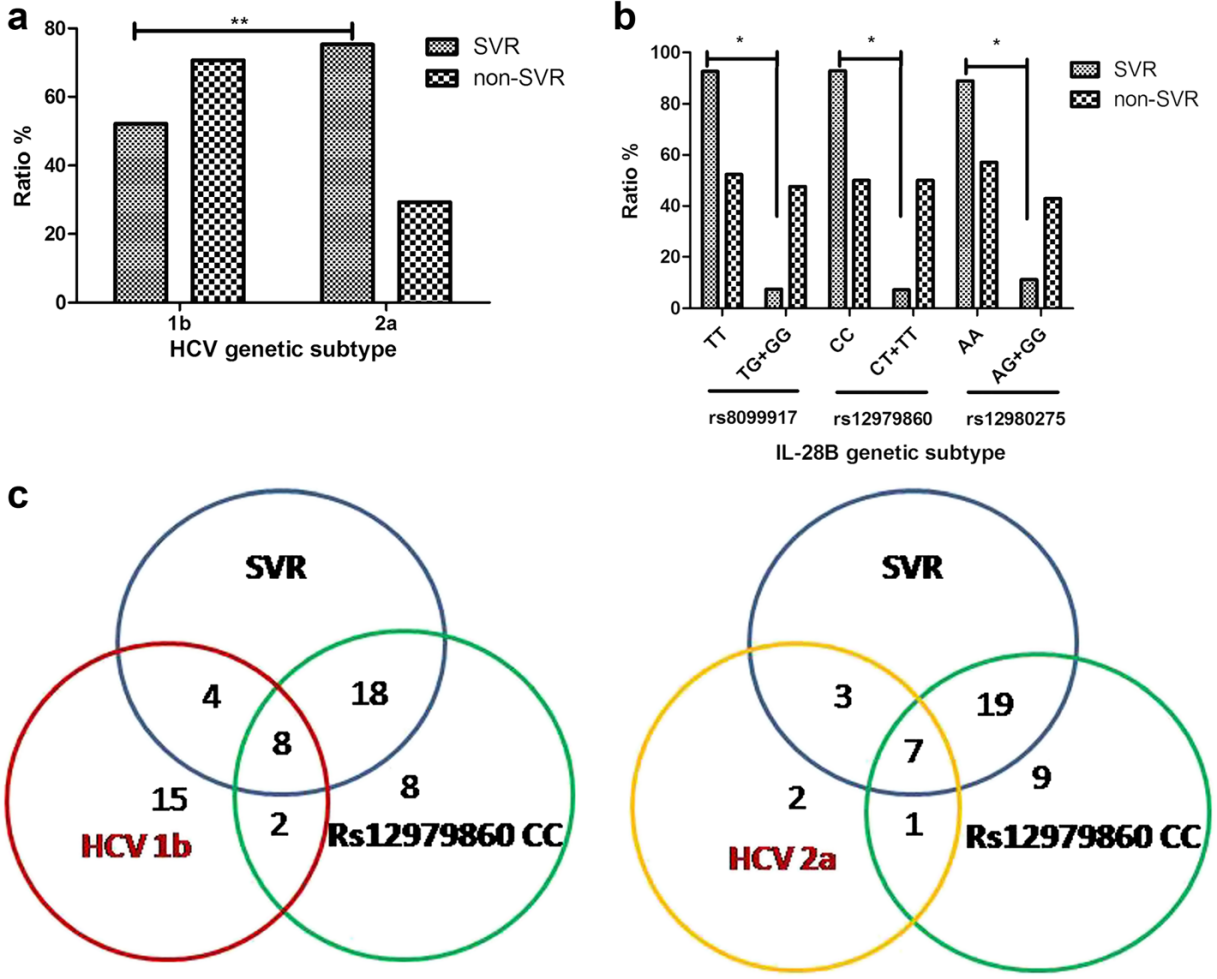
**a** Correlation between HCV genotype distribution and SVR. Sustained viral response (SVR) rates by HCV genotypes 1b and 2a were 52.25% (58/111) and 75.28% (67/89), statistically significant at *P* < 0.01. Rates of patients with HCV genotype 1b and 2a who did not reach SVR were 70.67 and 29.33%. Relationship between IL-28B genotype and SVR was showed in (**b**). SVR rates in patients of chronic HCV infection with different IL-28 single-nucleotide polymorphisms (SNP) (rs8099917 TT, rs12979860 CC and rs12980275 AA) were 92.41% (25/27), 92.86% (26/28), and 88.89% (24/27), respectively. In contrast, only 7.41, 7.14 and 11.11% of patients with IL-28B rs8099917 TG + GG, rs12979860 CT + TT, and rs12980275 AG + GG obtained SVR. **c** showed correlation among IL-28B SNP, HCV genotype and SVR

HCV genotypes 1b and 2a accounted for 86% of all samples; therefore, we mainly evaluated the correlation between these 2 genotypes and antiviral efficacy. SVR rates in patients with HCV genotype 1b and HCV genotype 2a were 52.25% (58/111) and 75.28% (67/89), with differences being statistically significant (chi-squared =21.56; *P* < 0.01). In patients with HCV genotype 1b, 70.67% (53/75) did not reach SVR (Fig. 2a).

### Role of IL-28B gene polymorphism in antiviral efficacy

We used SNP Stats Software (http://bioinfo.iconcologia.net/snpstats/start.htm [13]) for correlation analyses. After adjusting for confounding factors (including age, sex, viral load, ALT and AST), we found that there was a correlation between patients with SVR and IL-28B genotype (*P* < 0.05), with OR (95% confidence interval) of11.02 (3.98–23.16) for rs8099917, 10.20 (5.23–22.14) for rs12979860 and 10.08 (2.98–18.59) for rs12980275. Figure 2b show that patients with chronic HCV infection the SVR rate of IL-28B rs8099917 TT was (25/27,92.41%), IL-28B rs12979860 CC was (26/28, 92.86%) and IL-28B rs12980275 AA was (24/27, 88.89%). In contrast, only 7.41, 7.14 and 11.11% reached SVR with IL-28B SNP rs8099917 TG + GG, rs12979860 CT + TT, and rs12980275 AG + GG, respectively. Patients with protective genotypes were more likely to obtain SVR.

### Role of IL-28B SNP and HCV genotype in antiviral efficacy

We combined the results of IL-28B SNP and HCV genotype and analyzed the correlation between these factors. We found that 12/51 patients with HCV genotype 1b infection obtained SVR, in which 8/12 cases were rs12979860 CC (shown in Fig. 2c). We also found that the 7 of10 patients infected with HCV genotype 2a who obtained SVR were rs12979860 CC. In addition, 2 in 17 patients with HCV genotype 1b infection and SNP rs12979860 CC did not obtain SVR, and only 1/3 patients with HCV genotype 2a infection and SNP rs12979860 CC did not obtain SVR. Patients HCV genotype 2a infection with IL-28B SNP rs12979860 CC were more likely to reach SVR.

### Association of liver fibrosis staging and cirrhosis in patients with chronic HCV infection and antiviral efficacy

Because there were only 190 out of 231 patients did PLT detection, and APRI and FIB-4 were calculated according to results of patients’ PLT. So APRI score and FIB-4 index were calculated in 190 of 231 patients with chronic HCV infection. Sixty patients had FIB-4 index > 3.25, including 32 patients with genotype 1b infection and 21 patients with genotype2a infection. In this patient group, 31.0% of patients with HCV genotype 1b (19/32 × 58/111) and 39.4% of patients with genotype 2a (11/21 × 67/89) reached SVR (not statistically significant; *P* > 0.05). Thirty-nine patients had APRI score > 2, including 11 patients with genotype 1b and 8 patients with genotype 2a infection. In this patient group, 30.3% with HCV genotype 1b (11/19 × 58/111) and 50.2% with genotype 2a (8/12 × 67/89) reached SVR (*P* < 0.05). Although we could not prove that genotype affected the ability to reach SVR in patients with cirrhosis (APRI > 2), we did observe that APRI score > 2 significantly affected SVR. Details are shown in Tables 4 and 5.

**Table 4:** Characteristics of patients

Variables	No. of Patients (%of Total)
Total number of patients	231
Age ± standard deviation, y	46.71 ± 14.34
Male	113
Female	118
APRI data	190 (82.3)
FIB-4 data	190 (82.3)

**Table 5:** FIB-4 index, APRI scores and SVR of patients

Genotype	No. of Patients (% of Total)	No. of Patients with FIB-4 > 3.25 (% of Total)	No. of Patients with APRI > 2 (% of Total)	Total SVR (%)	No. of Patients with FIB-4 > 3.25 and SVR (%)	No. of Patients with APRI > 2 and SVR (%)
1b	90 (81.1)	32 (16.8)	19 (10.0)	58 (64.4)	19 (31.0)	11 (30.3)
2a	76 (85.4)	21 (11.1)	12 (6.3)^*^	67 (88.1)	11 (39.4)	8 (50.2)
Other	24 (77.4)	7 (3.7)	8 (4.2)	13		
Total	190 (82.3)	60 (31.6)	30 (20.5)	138 (72.6)		

^*^ *P* < 0.05

## Discussion

China has shown a new trend in HCV infection, with epidemic levels of genotypes1, 2, 3 and 6 and no genotypes 4 or 5 found. The most common genotype in China is1b and 2a, with incidence rates of 73.1 and 18.5%, followed by genotypes 3a, 6a, 3b and 1a. Genotypes 3 and 6 are geographically distributed more widely [20]. In our group, which included 230 patients with chronic HCV infection, sequencing results showed that the incidence of HCV in the Shaanxi region was > 86% with genotypes 1 and 2, with much lower numbers with genotypes 3 and 6, and none with genotype 4. More patients had genotype 1b (48.05%) and 2a (38.53%), followed by 1a, 3a, 3b, 2b and 6a. The high percent of genotype 2a might be one important reason for high SVR. And we truly found that patients with genotype 2a had greater SVR rates (75.28%) than patients with genotype 1b (52.25%). The results have a slight discrepancy versus the previous reports, which SVR rates for patients with genotype 1 were 42–46% and non-type 1 genotype were 76–82% [10, 21–23].

Furthermore, our SNP analysis results of 51 patients with chronic HCV infection (IL-28B rs8099917, IL-28B rs12979860 and IL-28B rs12980275 SNP) and sequencing results of 230 patients with chronic HCV infection showed that more patients had IL-28B rs8099917 TT versus rs8099917 GG, more had IL-28B rs12979860 CC versus TT, and more had IL-28B rs12980275 AA versus GG in Northwest China, similar to some previous reports [24, 25]. Presence of rs8099917 is one of the independent predictors in HCV 1b patients treated with PEG-IFN-α/R or interferon-α 2 only. Presence of rs12980275 has great relevance with SNP of rs12979860 [26], which plays an important role in the prediction of SVR in HCV patients treated with PEG-IFN-α/R, particularly in patients with HCV genotype 1 or 4 [10, 27]. So rs8099917TT, rs12979860CC, and rs12980275AA were considered to be protective genotypes [28]. In this study, SVR rates of patients with IL-28B rs8099917 TT, rs12979860 CC and rs12980275 AA were 92.41% (25/27), 92.86% (26/28) and 88.89% (24/27) separately. SVR rates in patients with protective genotypes accounted for more than sixty percent of total, whereas the SVR rates of non-protective genotypes were very low. This suggested that the protective IL-28B genotypes were more likely to result in patients with chronic HCV infection obtaining SVR, which is consistent with previous reports [26–29]. In addition, we corrected for age, sex, AST and ALT levels, HCV genotype and other factors with the use of SNPStats software [30] and found that patients with chronic HCV infection who obtained SVR 24 weeks after standard of care antiviral treatment were closely related to HCV genotype and IL-28B SNP (*P* < 0.05). That means more than sixty percent of patients are suitable for PEG-IFN-α/R treatment. Well, the major limitation in this part of study is that the sample amount was relatively too small to determine the role of IL-28B gene polymorphism in antiviral efficacy. However, our findings were in accordance with previous data [26–29].

Interestingly, we found that differences in cirrhosis progression between 1b and 2a were statistically significant, confirming that HCV genotype can influence the progression of liver cirrhosis. FIB-4 index > 3.25 or APRI score > 2 indicated that patients had significant liver fibrosis or even cirrhosis [31]. In this study, the SVR rates of patients with FIB-4 index > 3.25 and genotypes1b and 2a were 31.0 and 39.4%, respectively, which were much lower than that shown in patients with genotype 1b (62.06%) and genotype 2a (74.54%) with FIB-4 index ≤ 3.25. This indicated that patients with obvious liver fibrosis were less likely to reach SVR, which was associated with HCV genotype (1b or 2a). In addition, SVR rates of patients with APRI score > 2 were 30.3% for genotype 1b and 50.2% for genotype 2a. The large difference indicated that patients with cirrhosis had greater difficulty reaching SVR, for both HCV genotype 1b and 2a, and the progression of cirrhosis (APRI > 2) could be influenced by genotype. The clearance of HCV in patients with advanced-stage liver fibrosis can reduce the incidence of decompensated liver cirrhosis.

## Conclusions

There were high proportion of HCV genotype 1b and 2a in Northwest China. In both HCV 1b and 2a genotypes, patients with protective-genotype of IL-28B were more likely to obtain SVR. However, those with significant fibrosis or cirrhosis were less likely, no matter their genotype. Combined factors of HCV genotype, IL-28B genotype, FIB-4 and ARPI potentially have a very high prediction and clinical value regarding treatment with PEG-IFN-α/R and prognostic evaluation of chronic hepatitis C patients.

## Abbreviations

APRI: Aspartate aminotransferase-to-platelet ratio index
CHC: Chronic hepatitis C
FIB-4: Fibrosis 4 index
HCV: Hepatitis C virus
IL-28B: Interleukin (IL)-28B
PEG-IFN-α/R: Pegylated interferon-alpha combined with ribavirin
SNP: Single-nucleotide polymorphisms
SVR: Sustained viral response

## References

[1] Antonelli A, Pistello M. New Therapies, Markers and Therapeutic Targets in HCV Chronic Infection, and HCV Extrahepatic Manifestations. Curr Drug Targets. 2015. 10.2174/1389450116666151102095708 26521772

[2] Gower E, Estes C, Blach S, Razavi-Shearer K, Razavi H (2014). Global epidemiology and genotype distribution of the hepatitis C virus infection. J Hepatol.

[3] Pellicelli AM, Romano M, Stroffolini T, Mazzoni E, Mecenate F, Monarca R, Picardi A, Bonaventura ME, Mastropietro C, Vignally P (2012). HCV genotype 1a shows a better virological response to antiviral therapy than HCV genotype 1b. BMC Gastroenterol.

[4] Gane EJ, Stedman CA, Hyland RH, Ding X, Svarovskaia E, Symonds WT, Hindes RG, Berrey MM (2013). Nucleotide polymerase inhibitor sofosbuvir plus ribavirin for hepatitis C. N Engl J Med.

[5] Lawitz E, Poordad FF, Pang PS, Hyland RH, Ding X, Mo H, Symonds WT, McHutchison JG, Membreno FE (2014). Sofosbuvir and ledipasvir fixed-dose combination with and without ribavirin in treatment-naive and previously treated patients with genotype 1 hepatitis C virus infection (LONESTAR): an open-label, randomised, phase 2 trial. Lancet.

[6] Chhatwal J, Kanwal F, Roberts MS, Dunn MA (2015). Cost-effectiveness and budget impact of hepatitis C virus treatment with sofosbuvir and ledipasvir in the United States. Ann Intern Med.

[7] Trinks J, Hulaniuk ML, Caputo M, Pratx LB, Re V, Fortuny L, Pontoriero A, Frias A, Torres O, Nunez F (2014). Distribution of genetic polymorphisms associated with hepatitis C virus (HCV) antiviral response in a multiethnic and admixed population. Pharmacogenomics J.

[8] Wu CK, Chang KC, Tseng PL, Lu SN, Chen CH, Wang JH, Lee CM, Lin MT, Yen YH, Hung CH, Hu TH (2016). Comparison of therapeutic response and clinical outcome between HCV patients with normal and abnormal alanine transaminase levels. PLoS One.

[9] Wang M, Zhang Y, Li Z, Zhang H, Zhang Z, Yue D, Zhou R, Li X, Wu S, Li J (2015). Hepatitis C virus (HCV) genotype 2a has a better virologic response to antiviral therapy than HCV genotype 1b. Int J Clin Exp Med.

[10] Liu T, Sha K, Yang L, Wang Y, Zhang L, Liu X, Yang F (2014). IL-28B polymorphisms correlated with treatment response in HCV-4 mono-infected patients: a meta-analysis. PLoS One.

[11] Mechie NC, Rover C, Cameron S, Amanzada A (2014). Predictability of IL-28B-polymorphism on protease-inhibitor-based triple-therapy in chronic HCV-genotype-1 patients: A meta-analysis. World J Hepatol.

[12] Morales-Sanchez A, Fuentes-Panana EM (2014). Human viruses and cancer. Viruses.

[13] Shindo H, Maekawa S, Komase K, Miura M, Kadokura M, Sueki R, Komatsu N, Shindo K, Amemiya F, Nakayama Y (2013). IL-28B (IFN-lambda3) and IFN-alpha synergistically inhibit HCV replication. J Viral Hepat.

[14] Yoshida H, Shiratori Y, Moriyama M, Arakawa Y, Ide T, Sata M, Inoue O, Yano M, Tanaka M, Fujiyama S (1999). Interferon therapy reduces the risk for hepatocellular carcinoma: national surveillance program of cirrhotic and noncirrhotic patients with chronic hepatitis C in Japan. IHIT Study Group. Inhibition of Hepatocarcinogenesis by Interferon Therapy. Ann Intern Med.

[15] Singal AG, Volk ML, Jensen D, Di Bisceglie AM, Schoenfeld PS (2010). A sustained viral response is associated with reduced liver-related morbidity and mortality in patients with hepatitis C virus. Clin Gastroenterol Hepatol.

[16] Wei L, Hou JL, Chinese Society of Hepatology CMA, Chinese Society of Infectious Diseases CMA (2015). The guideline of prevention and treatment for hepatitis C: a 2015 update. Zhonghua Gan Zang Bing Za Zhi.

[17] Hou JL, Chinese Society of Infectious Diseases CMA (2015). The guideline of prevention and treatment for chronic hepatitis B: a 2015 update. Zhonghua Gan Zang Bing Za Zhi.

[18] Lens S, Torres F, Puigvehi M, Marino Z, Londono MC, Martinez SM, Garcia-Juarez I, Garcia-Criado A, Gilabert R, Bru C (2016). Predicting the development of liver cirrhosis by simple modelling in patients with chronic hepatitis C. Aliment Pharmacol Ther.

[19] Andres-Otero MJ, De-Blas-Giral I, Puente-Lanzarote JJ, Serrano-Aullo T, Morandeira MJ, Lorente S, Lou-Bonafonte JM (2016). Multiple approaches to assess fourteen non-invasive serum indexes for the diagnosis of liver fibrosis in chronic hepatitis C patients. Clin Biochem.

[20] Gu L, Tong W, Yuan M, Lu T, Li C, Lu L (2013). An increased diversity of HCV isolates were characterized among 393 patients with liver disease in China representing six genotypes, 12 subtypes, and two novel genotype 6 variants. J Clin Virol.

[21] Waldenstrom J, Farkkila M, Rembeck K, Norkrans G, Langeland N, Morch K, Pedersen C, Rauning Buhl M, Nieminen U, Nuutinen H (2016). Short interferon and ribavirin treatment for HCV genotype 2 or 3 infection: NORDynamIC trial and real-life experience. Scand J Gastroenterol.

[22] Tamori A, Yoshida K, Kurai O, Kioka K, Hai H, Kozuka R, Motoyama H, Kawamura E, Hagihara A, Uchida-Kobayashi S (2016). Randomized trial of combined triple therapy comprising two types of peginterferon with simeprevir in patients with HCV genotype 1b. Hepatol Res.

[23] Deming P, Martin MT, Chan J, Dilworth TJ, El-Lababidi R, Love BL, Mohammad RA, Nguyen A, Spooner LM, Wortman SB (2016). Therapeutic advances in HCV genotype 1 infection: insights from the society of infectious diseases pharmacists. Pharmacotherapy.

[24] Ban JY, Yoo KH (2014). Promoter polymorphism (rs12770170, −184C/T) of microseminoprotein, beta as a risk factor for benign prostatic hyperplasia in Korean population. Int Neurourol J.

[25] Utsumi T, Lusida MI (2015). Viral hepatitis and human immunodeficiency virus co-infections in Asia. World J Virol.

[26] Shaker O, Rashad A, Abd El Aziz G, El Raziky M (2015). Is rs8099917 polymorphism of IL-28B gene a good predictor of response to therapy of HCV than rs12979860? An Egyptian study. Cell Biochem Biophys.

[27] Khubaib B, Saleem S, Idrees M, Afzal S, Wasim M (2015). The genotype CC of IL-28B SNP rs12979860 is significantly associated with a sustained virological response in chronic HCV-infected Pakistani patients. J Dig Dis.

[28] Al-Qahtani A, Al-Anazi M, Abdo AA, Sanai FM, Al-Hamoudi W, Alswat KA, Al-Ashgar HI, Khan MQ, Albenmousa A, Khalaf N (2015). Correlation between genetic variations and serum level of interleukin 28B with virus genotypes and disease progression in chronic hepatitis C virus infection. J Immunol Res.

[29] Tanaka Y, Nishida N, Sugiyama M, Kurosaki M, Matsuura K, Sakamoto N, Nakagawa M, Korenaga M, Hino K, Hige S (2009). Genome-wide association of IL28B with response to pegylated interferon-alpha and ribavirin therapy for chronic hepatitis C. Nat Genet.

[30] Wedemeyer H (2015). Towards interferon-free treatment for all HCV genotypes. Lancet.

[31] Kogiso T, Tokushige K, Hashimoto E, Ikarashi Y, Kodama K, Taniai M, Torii N, Shiratori K (2016). Safety and efficacy of long-term tolvaptan therapy for decompensated liver cirrhosis. Hepatol Res.

